# Evidence for a Retroviral Insertion in *TRPM1* as the Cause of Congenital Stationary Night Blindness and Leopard Complex Spotting in the Horse

**DOI:** 10.1371/journal.pone.0078280

**Published:** 2013-10-22

**Authors:** Rebecca R. Bellone, Heather Holl, Vijayasaradhi Setaluri, Sulochana Devi, Nityanand Maddodi, Sheila Archer, Lynne Sandmeyer, Arne Ludwig, Daniel Foerster, Melanie Pruvost, Monika Reissmann, Ralf Bortfeldt, David L. Adelson, Sim Lin Lim, Janelle Nelson, Bianca Haase, Martina Engensteiner, Tosso Leeb, George Forsyth, Michael J. Mienaltowski, Padmanabhan Mahadevan, Michael Hofreiter, Johanna L. A. Paijmans, Gloria Gonzalez-Fortes, Bruce Grahn, Samantha A. Brooks

**Affiliations:** 1 Department of Biology, University of Tampa, Tampa, Florida, United States of America; 2 Department of Animal Science, Cornell University, Ithaca, New York, United States of America; 3 Department of Dermatology, School of Medicine and Public Health, University of Wisconsin, Madison, Wisconsin, United States of America; 4 Quill Lake, Saskatchewan, Canada; 5 Department of Small Animal Clinical Sciences, Western College of Veterinary Medicine, University of Saskatchewan, Saskatoon, Saskatchewan, Canada; 6 Department of Evolutionary Genetics, Leibniz Institute for Zoo and Wildlife Research, Berlin, Germany; 7 Epigenomic and Palaeogenomic Group, Institut Jacques Monod, Paris, France; 8 Department of Breeding Biology and Molecular Genetics, Humboldt University Berlin, Berlin, Germany; 9 School of Molecular and Biomedical Science, the University of Adelaide, South Australia, Australia; 10 Faculty of Veterinary Science, University of Sydney, Sydney, New South Wales, Australia; 11 Institute of Genetics, University of Bern, Bern, Switzerland; 12 Department of Veterinary Biomedical Sciences, Western College of Veterinary Medicine, University of Saskatchewan, Saskatoon, Saskatchewan, Canada; 13 Department of Molecular Pharmacology & Physiology, College of Medicine, University of South Florida, Tampa, Florida, United States of America; 14 Department of Biology, The University of York, York, United Kingdom; University of Iowa, United States of America

## Abstract

Leopard complex spotting is a group of white spotting patterns in horses caused by an incompletely dominant gene (*LP*) where homozygotes (*LP/LP*) are also affected with congenital stationary night blindness. Previous studies implicated *Transient Receptor Potential Cation Channel, Subfamily M, Member 1* (*TRPM1*) as the best candidate gene for both CSNB and *LP*. RNA-Seq data pinpointed a 1378 bp insertion in intron 1 of *TRPM1* as the potential cause. This insertion, a long terminal repeat (LTR) of an endogenous retrovirus, was completely associated with *LP*, testing 511 horses (χ^2^=1022.00, p<<0.0005), and CSNB, testing 43 horses (χ^2^=43, p<<0.0005). The LTR was shown to disrupt *TRPM1* transcription by premature poly-adenylation. Furthermore, while deleterious transposable element insertions should be quickly selected against the identification of this insertion in three ancient DNA samples suggests it has been maintained in the horse gene pool for at least 17,000 years. This study represents the first description of an LTR insertion being associated with both a pigmentation phenotype and an eye disorder.

## Introduction

Domestic mammals are selectively bred for variability in coat color and pattern, making pigmentation an attractive system to study. In addition, mutant genes controlling pigmentation in both domestic and experimental mammals have been shown to have pleotropic effects leading to vision, hearing, and neuronal deficits, among others, and have become important means to study human disorders [1-5].

**Figure 1:**
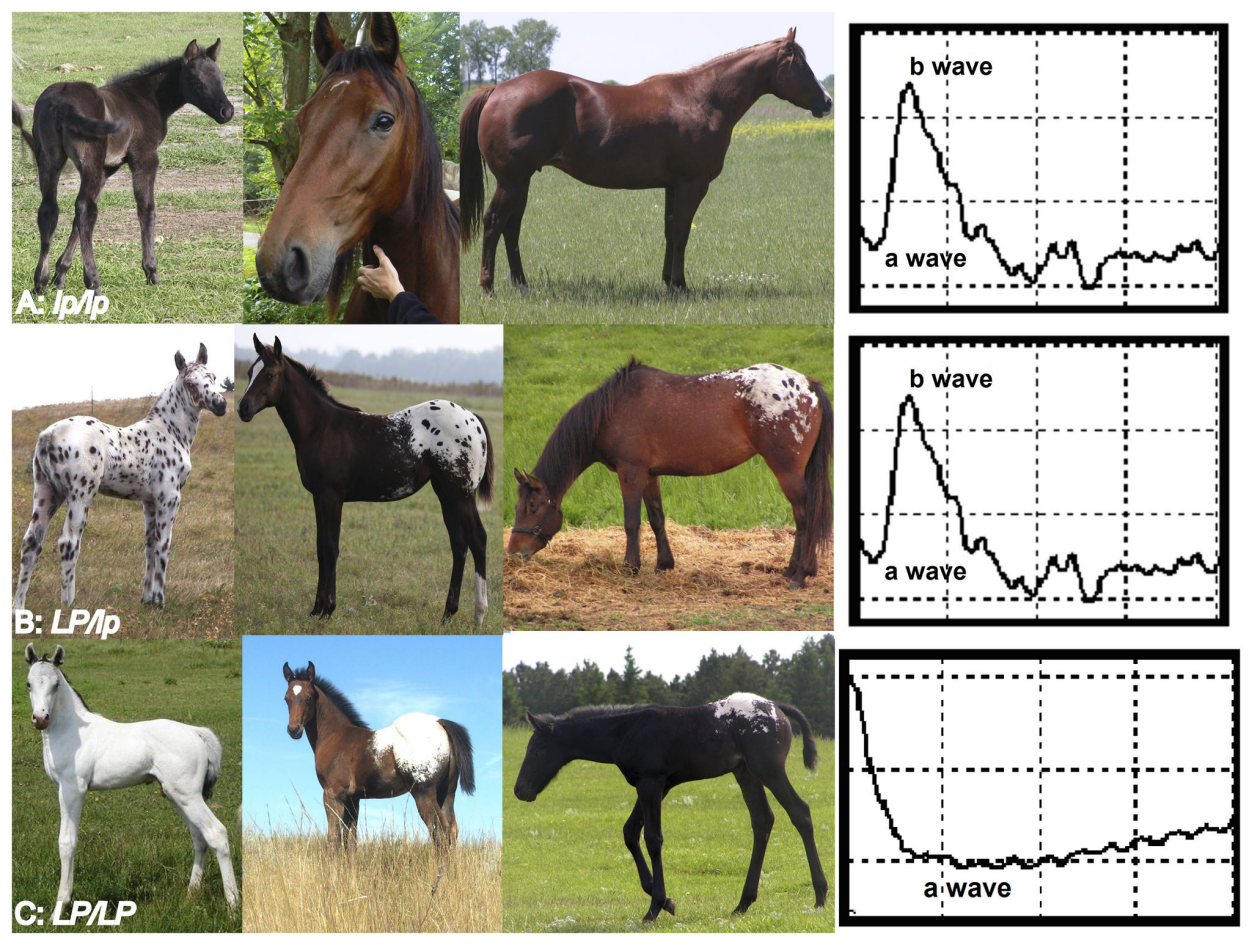
Congenital stationary night blindness is associated with homozygosity for leopard complex spotting in horses. (A) *lp/lp* horses do not display a leopard complex spotting pattern and are not night blind as evidenced by normal ERGs. (B) *LP/lp* horses display one of several characteristic spotting patterns that vary in the amount of un-pigmented hairs in the coat and have pigmented spots (“leopard spots”) in the un-pigmented area. These horses are not night blind as evidenced by normal ERGs. (C) *LP/LP* horses displaying the characteristic homozygous patterns with varying amounts of white on the coat with few to no “leopard spots”. These horses all have CSNB as evidenced by the “negative” ERG in which the b wave is absent.

Complete congenital stationary night blindness (CSNB) is one such example, where discovery of causal mutations in humans was aided by pigmentation studies in the horse [6-9]. Specifically, horses homozygous for a white spotting phenotype, known as leopard complex spotting, are affected by CSNB [10,11]. CSNB is characterized by a congenital and non-progressive impaired vision in dark condition. It is diagnosed by dark adapted “negative” electroretinography (ERG) where the b-wave (arising from ON-bipolar cell activation) is absent and the a-wave has increased amplitude (arising from photo receptor activation; Figure 1) [12,13]. 

**Figure 2:**
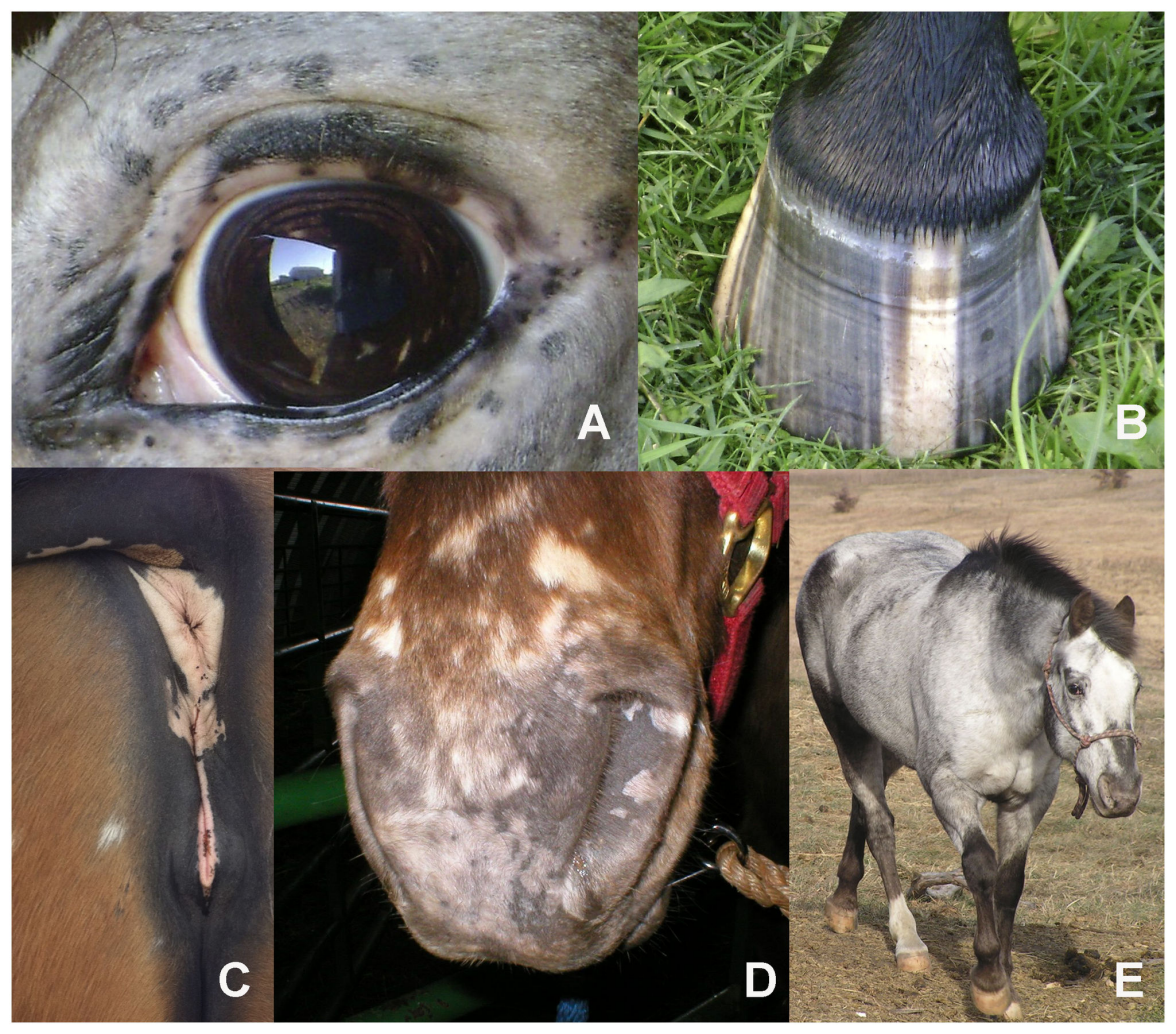
*LP*-associated characteristics. In addition to coat color spotting patterns *LP* also causes four other associated pigmentation characteristics, readily visible white sclera (as shown in A) striped hooves, which are bands of pigment on the hoof alternating with unpigmented bands (as shown in B), mottling, which is pink skin with spots of pigment that occurs around the anus, genitalia (C), muzzle (D), and eyes (E), and varnish roaning, the progressive loss of pigment throughout the coat while retaining pigment on boney surfaces; as shown here on the face, hips, and lower legs (E).

Leopard complex spotting (*LP*) is found in several breeds of horse and is characterized by the absence of pigment (white spotting) in the coat (Figure 1B and C), and associated pigmentation characteristics (Figure 2) [14]. These white spotting patterns tend to be symmetrical and centered over the hips. However, the amount of white on the coat varies and can range from a few white flecks on the rump to horses that are almost completely white (Figure 1B and C). *LP* associated characteristics include white sclera, striped hooves, mottling, and progressive roaning, known as varnish roan, as shown in Figure 2. While all horses, like other mammals have a sclera, the white surrounding the eyeball, in *LP* horses the sclera is more visible due to a lack of perilimbal bulbar conjunctival pigmentation. *LP* spotted horses also have vertical bands of pigment on their hooves known as stripped hooves. Mottling is characterized by pink skin with patches of pigmented skin occurring around the eye, muzzle, anus, and genitalia of the horse. Finally varnish roaning is the progressive loss of pigment in the coat, as the horse ages, while maintaining full pigment on the bony surfaces of the face, hips, and, lower legs. While these *LP* associated characteristics all appear to result from a reduction in pigmentation, work is ongoing to determine if these characteristics are caused by a reduction in melanocyte number or loss of melanocyte function. 

Leopard complex spotting is inherited as a single incompletely dominant trait (*LP*), where homozygotes in addition to being night blind have few or no spots of pigment known as “leopard spots” (Figure 1C) [14,15]. *LP* was mapped to ECA 1; positional and functional candidate genes were investigated by qRT-PCR and *transient receptor potential cation channel subfamily M member 1* (*TRPM1*) was implicated as the cause for both CSNB and *LP* [16,17]. Following this discovery, investigation of human complete-CSNB with no known genetic cause led to the identification of mutations in *TRPM1* as the most frequent causal mutations of autosomal recessive CSNB [6-9]. Concurrently, work in mice localized TRPM1 to the ON-bipolar cell dendrites and identified TRPM1 as the non-selective ion channel that generates the depolarizing current in ON bipolar cells [18-21]. In terms of pigmentation*, TRPM1* was first identified in melanoma cells as RNA levels were inversely correlated with melanoma progression and tumor thickness [22] and has since been correlated with melanin content, and implicated in pigment storage and UV regulated calcium homeostasis in human melanocytes [23,24]. TRPM1 therefore has potential roles in Ca^2+^-dependent signaling related to melanocyte proliferation, differentiation, and/or survival.

DNA sequencing of annotated *TRPM1* exons did not identify a causative mutation, however fine mapping detected a single 173 kb haplotype (ECA1: 108,197,355- 108,370,150) associated with *LP* and CSNB [25]. Targeted DNA re-sequencing of a 300 kb region surrounding this haplotype in two homozygotes (one Knabstrupper and one Appaloosa; two breeds famous for their leopard complex spotting patterns) identified six polymorphisms near this gene for further investigation. Three of these were perfectly associated with *LP* phenotype in 7 breeds of horses and with CSNB in 2 breeds (ECA1 g.108281765TC, ECA1 g.108288853CT, and ECA1g.108337089TG) [11,25,26]. Additionally, splice variants identified two novel exons in human *TRPM1* (termed exon 0 and 1’) [23]. Therefore, we utilized RNA-Seq technologies to investigate the hypothesis that these three associated SNPs were within unannotated *TRPM1* exons in the horse and to determine if these or other mutations identified by RNA-Seq were causative for *LP* and CSNB. Furthermore, it was unknown if *LP* pigmentation characteristics are caused by absence of melanocytes or non-functioning melanocytes. Thus, here we also sought to isolate, culture, and characterize epidermal melanocytes in *LP/LP* and *lp/lp* horses.

## Results

### RNA-sequencing

**Figure 3:**
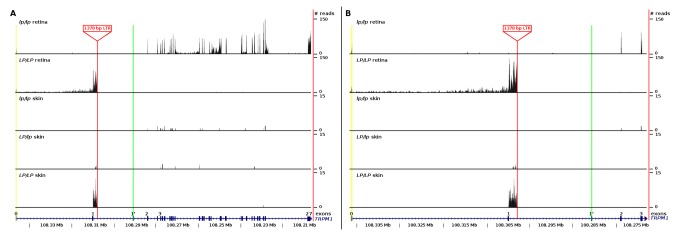
RNA sequencing reads mapped to *TRPM1*. RNA-Seq reads from *LP/LP* CSNB affected and *lp/lp* CSNB unaffected retina RNA, as well as, from *LP/LP, LP/lp* and *lp/lp* skin RNA. ECA1 Mb*, TRPM1* exon position, location of the insertion, and the number of reads for each sample are presented. (A) exon 0-27 illustrating that in *LP/LP* samples transcripts were not detected in exons 3’ of the insertion in intron 1. (B) exon 0, 1, 1’, 2 and 3 of TRPM1 illustrating that transcripts from exon 0 (yellow highlight) were detected in retinal samples but not skin while transcripts from exon 1’ (green highlight) were not detected in any of the horse samples, and finally transcripts were detected that mapped to intron 1 of *LP/LP* and *LP/lp* samples allowing for the identification of the insertion (denoted by red line).

Illumina RNA-Seq from five samples (three from skin and two from retina) generated over 43,000,000 reads of which an average of 69.32 % of these reads mapped to the EquCab2 genome assembly. Summary statistics for this sequencing can be found in Table 1. RNA sequencing determined that the three associated SNPs were not detected in a yet uncharacterized exon in horse *TRPM1*, as no reads were identified covering these SNPs in the five horses sequenced (Figure S1). Both ECA1 g.108281765TC (Figure S1A) and ECA1 g.108288853CT (Figure S1B) are located in introns of *TRPM1* (as defined by human, rat and mouse RefSeq genes) and were not detected in RNA-Seq reads from either skin or retina. ECA1g.108337089TG (Figure S1C) is also not in a transcribed region, as no expression was detected and no known RefSeq gene has been mapped to this region. From these data we also determined that exon 0, detected in human melanocyte, brain, and retina [23] was also present in the horse retina samples but not in skin (Figure 3). Additionally, exon 1’, expressed at low levels in human melanocytes, brain, and retina (<2.5, 5, and 4 % respectively) [23], was not detected in either skin or retina of the horse (Figure 3).

**Table 1:** Summary of Illumina RNA-Seq data from the 5 samples used to identify the causative structural variant for *LP* and CSNB.

***RNASample***	***Reads***	***% Mapped***	***Length***
*LP/LP* pigmented skin	7,982,171	*66.02*	78 bp
*lp/lp* pigmented skin	7,111,639	*62.25*	78 bp
*LP/lp* pigmented skin	8,392,169	*72.13*	81 bp
CSNB affected retina	10,261,636	*66.14*	81 bp
CSNB Unaffected retina	10,109,154	*80.08*	81 bp
***Totals***	43,856,769		

### Polymorphism Detection and Analysis

**Figure 4:**
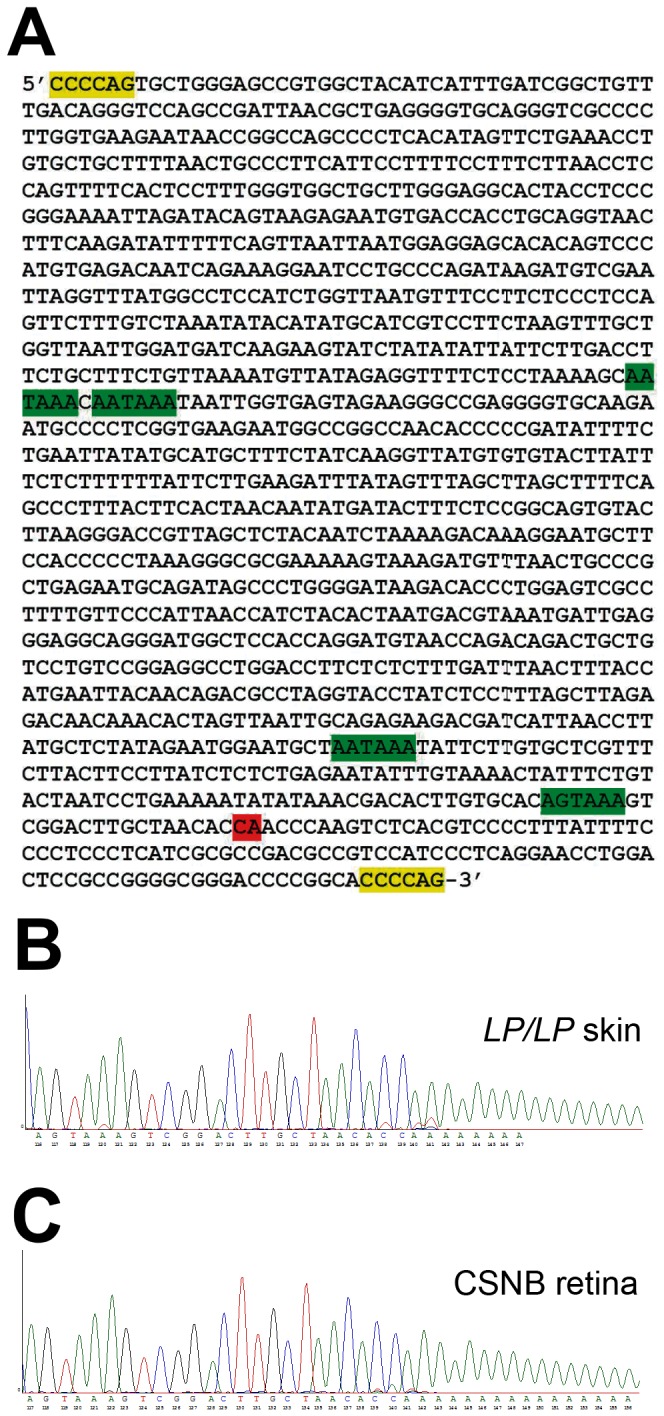
LTR insertion in *TRPM1* causes premature poly-adenylation. (A) The 1378bp LTR insertion (ECA1g. 108,297,929_108,297,930 ins1378) is flanked by 6 bp regions of micro-homology which are highlighted in yellow. Poly-adenylation signal sequences are highlighted in green. The CA dinucleotide cleavage and poly-adenylation site is highlighted in red. (B) poly(A) tail detected by 3’RACE in *LP/LP* skin and (C) CSNB *LP/LP* retina samples. Sequence represented is from poly-adenylation signal sequence AGUAAA through poly(A) tail.

Previous gene expression analyses [17] did not detect expression of *TRPM1* in the skin and retina of *LP/LP* CSNB individuals, suggesting that transcription of this gene was disrupted. However, when comparing raw read counts, we identified novel expression within intron 1, having 85 reads in the skin and 460 reads in the retina of the *LP/LP* horse compared to 0 and 4 reads (in the skin and retina respectively) of the *lp/lp* horse (Figure 3). *De novo* assembly of this transcript followed by PCR and genomic DNA sequencing identified a 1378 bp insertion in intron 1 (ECA1g. 108,297,929_108,297,930 ins1378) of *TRPM1*. In order to characterize this insertion we performed a blast analysis using RepeatMasker [27] (Figure S2). This insertion is a long terminal repeat (LTR) of an endogenous retrovirus (ERV) class II retro-element and is characterized by flanking regions of micro-homology, indicative of non-homologous end joining double stranded break repair as the mechanism of insertion (Figure 4A and Figure S2). Detection of this insertion by allele specific PCR confirmed complete association with the *LP* genotype (as predicted by phenotype) in tests of 511 horses (χ^2^=1022.00, p<<0.0005) and CSNB status in tests of 43 horses (χ^2^=43, p<<0.0005) (Table 2). 

**Table 2:** Complete association between CSNB, leopard complex spotting phenotype, and 1.4 kb insertion in intron 1 of *TRPM1*.

		Genotype for Insertion		
Breed	Phenotype/Genotype	I/I	I/-	-/-
**CSNB**				
Appaloosa	CSNB	13	0	0
	Unaffected	0	6	6
American Miniature Horse	CSNB	3	0	0
	Unaffected	0	5	4
Knabstrupper	CSNB	1	0	0
	Unaffected	0	1	0
Quarter Horse*	CSNB	0	0	0
	Unaffected	0	0	4
**Total (N=43) χ^2^=57, p<<0.0005**	**CSNB**	**17**	**0**	**0**
	**Unaffected**	**0**	**12**	**14**
***LP***				
Appaloosa	*LP^LP^/LP^LP^*	67	0	0
	*LP^LP^/LP^lp^*	0	84	0
	*LP^lp^/LP^lp^*	0	0	29
Knabstrupper	*LP^LP^/LP^LP^*	27	0	0
	*LP^LP^/LP^lp^*	0	32	0
	*LP^lp^/LP^lp^*	0	0	5
Noriker	*LP^LP^/LP^LP^*	2	0	0
	*LP^LP^/LP^lp^*	0	58	0
	*LP^lp^/LP^lp^*	0	0	52
American Miniature Horse	*LP^LP^/LP^LP^*	20	0	0
	*LP^LP^/LP^lp^*	0	32	0
	*LP^lp^/LP^lp^*	0	0	11
Pony of the Americas	*LP^LP^/LP^LP^*	4	0	0
	*LP^LP^/LP^lp^*	0	14	0
	*LP^lp^/LP^lp^*	0	0	2
British Spotted Pony	*LP^LP^/LP^LP^*	5	0	0
	*LP^LP^/LP^lp^*	0	19	0
	*LP^lp^/LP^lp^*	0	0	1
Australian Spotted Pony	*LP^LP^/LP^LP^*	1	0	0
	*LP^LP^/LP^lp^*	0	9	0
	*LP^lp^/LP^lp^*	0	0	0
Thoroughbred*	*LP^LP^/LP^LP^*	0	0	0
	*LP^LP^/LP^lp^*	0	0	0
	*LP^lp^/LP^lp^*	0	0	37
**Totals (N=511**)** χ^2^=1022.00, p<<0.0005**	***LP^LP^/LP^LP^***	**126**	**0**	**0**
	***LP^LP^/LP^lp^***	**0**	**248**	**0**
	***LP^lp^/LP^lp^***	**0**	**0**	**137**

* breeds not segregating for the *LP* mutation; I, presence of insertion; - absence of insertion

ERVs are remnants of infectious retroviruses that integrated into the genome and are now stably inherited. ERV LTRs have a central role in viral replication, integration, and in gene expression as they contain transcriptional regulatory elements [28-31]. Novel transcription, in the vicinity of the insertion, supports use of this LTR as alternative promoter in *LP* horses as normalized RPKM from the retina samples showed 131 times greater coverage surrounding the insertion than in the 5’ exons. However, lack of *TRPM1* exon expression downstream of the LTR insertion was evident in both skin and retina *LP* samples (Figure 3A), consistent with previous qRT-PCR data [17], indicating that this LTR does not cause *LP* and CSNB by acting as an alternative promoter. Instead, we hypothesized that because this LTR insertion contains four canonical poly-adenylation signal sequences (3 AAUAAA and 1 AGUAAA; Figure 4A) [28], it disrupts normal *TRPM1* expression by premature poly-adenylation, similar to a mechanism demonstrated in mouse endogenous retroviruses [32]. 3’ RACE followed by sequencing of RNA from *LP/LP* CSNB affected samples supports the use of one of the poly-adenylation signals (AGUAAA) 19 nucleotides from a novel poly(A) (Figure 4B-C). Thus, premature poly-adenylation explains the dramatic loss of *TRPM1* expression 3’ of the insertion (Figure 3). 

Bioinformatic analysis of the LTR insertion transcript identified nine possible open reading frames with the longest predicted protein being 90 amino acids. Performing a BLAST search with these ORFs (http://blast.ncbi.nlm.nih.gov/Blast.cgi) did not identify any putative conserved domains or proteins with significant homology. However predicting protein function of the translated ORFs using the PFP and ESG servers [33,34] yielded several putative Gene Ontology (GO) molecular functions and biological processes (Table S1). For the longest ORF (90 amino acids) the PFP server predicted a molecular function of protein kinase CK2 activity with a probability of 83% and a biological process of regulation of retinal programmed cell death with a probability of 80%, among others. The ESG server did not produce predictions greater than the cutoff of 60% probability. For the second longest ORF (70 amino acid), PFP predicted a molecular function of dolichyl-phosphate beta-D-mannosyltransferase activity with a probability of 90% and a biological process of cofactor metabolism with a probability of 82%, among others. The ESG server predicted a molecular function of RNA binding for this ORF with a probability of 60% and a biological process of RNA-dependent DNA replication with a probability of 60%. 

Investigating the LP/CSNB associated haplotype region (ECA1: 108,197,355- 108,370,150) for additional variants in the *LP* samples identified four SNPs (Table 3). Two of these SNPs were previously identified (ECA1g.108, 298, 921A>T and ECA1g.108, 309,573T>G) [25] while two additional SNPs are novel discoveries (ECA1g.108,299,069GA and ECA1g.108,308,005CA). The regions harboring the SNPs were not expressed in the *lp/lp* samples suggesting that they are specific to the LTR insertion transcript resulting from premature poly-adenylation and were therefore not investigated further. 

**Table 3:** Additional polymorphisms identified by RNA-Seq in the LP/CSNB 173 kb associated haplotype.

	Number of reads and nucleotide detected				
Polymorphism	*lp/lp* skin	*LP/lp* skin	*LP/LP* skin	*lp/lp* retina	*LP/LP* retina
ECA1g. 108,298,921A>T	0	1T	4T	0	9T
ECA1g. 108,299,069G>A*	0	3A	2A	0	24A
ECA1g. 108,308,005C>A*	0	0	0	0	7A
ECA1g. 108,309,573T>C	0	0	0	0	5C

Number of reads and SNP calls for each sample are presented. * denotes novel SNPs discovered by this study.

### Characterizing the LTR in the horse genome

Comparison of the *LP* LTR sequence against all known LTR sequences in the horse revealed high sequence homology across the LTRs (Figure S3). Therefore, with these data it is not possible to calculate an exact age as most of these LTR are highly identical in sequence (172/197 having 94% or better identity). To gain a better approximation of dating the insertion event, eight ancient DNA samples that either previously typed positive for a SNP associated with *LP* [35] or were considered to be well preserved specimens were genotyped for this LTR insertion using single molecule capture coupled to Illumina sequencing (Table 4). Genotyping for large insertions in ancient DNA is difficult due to poor quality of the DNA, however here we used NGS technologies to identify the presence of this insertion. The LTR insertion was definitively detected in three samples. One of these samples dates to 15-14,000 BC (Table 4 and S2).

**Table 4:** Details of ancient DNA samples and LTR insertion typing results detected by MiSeq technologies.

**Sample**	**Origin**		**Date**	*TRPM1* Genotyping Results*	
	Excavation	Country			
KG5_1	Kniegrotte	Germany	15-14000 BC		L=30 bp**
874/11_1	Krasnokamenka	Russia	Neolithic		L=10 bp
874/6_1	Krasnokamenka	Russia	Neolithic		L=30 bp**
752/63_1	Lebyazhinka IV	Russia	Copper Age		L=10 bp
1137/16_1	Belokaragay	Russia	Copper Age		L=10 bp
1427/41_1	Alexandrovskoe IV	Russia	4300 BC		L=30 bp**
1427/59_1	Alexandrovskoe IV	Russia	4280 BC		L=10 bp
MAY10_1	Mayaki	Ukraine	2770 ± 40 BC		L=10 bp

DNA from each sample was extracted and amplified at least twice. * TRPM1 LTR insert detection based on single molecule capture and Illumina sequencing. For each sample the longest read length mapped to the probed sequence are presented. ** Mapped reads with length at least 30 bp (L=30 bp) was considered as conclusive evidence for the presence of the insertion.

### Melanocyte Cultures

**Figure 5:**
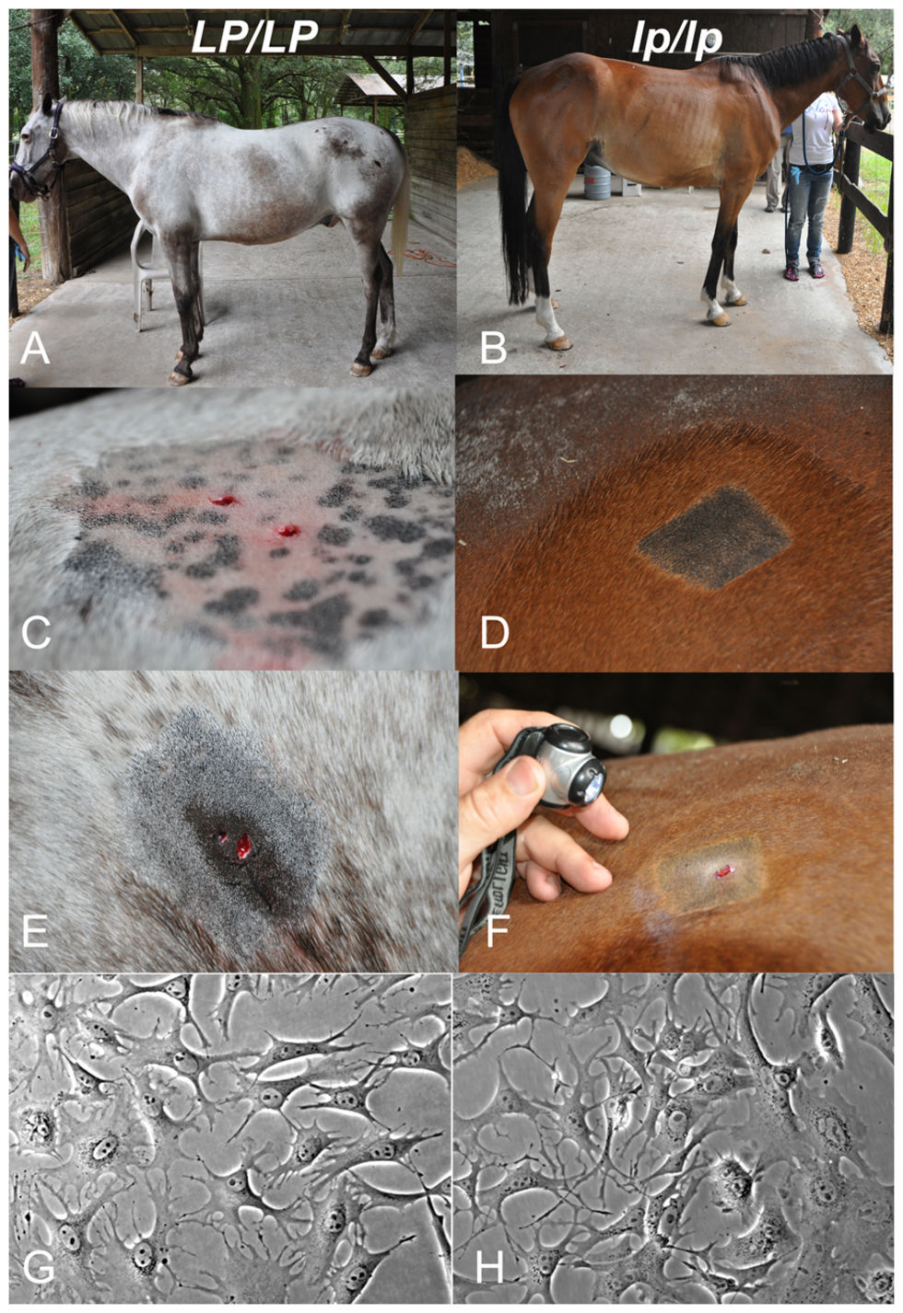
Skin biopsies and melanocyte cultures for *LP/LP* and *lp/lp* samples. Coat color phenotypes of (A) *LP/LP* horse and (B) *lp/lp* . Close-up views of the biopsied skin from *LP/LP* horse (C) unpigmented samples and (E) pigmented spot. Close up view of (D) pigmented area and (F) biopsied skin from *lp/lp* horse taken from approximately the same location as the *LP/LP* pigmented spot on the rump. Morphology of (G) *LP/LP* and (H) *lp/lp* melanocytes in culture at passage 7 and 6 respectively. Culture images were captured on Nikon microscope with 20x objective.

**Figure 6:**
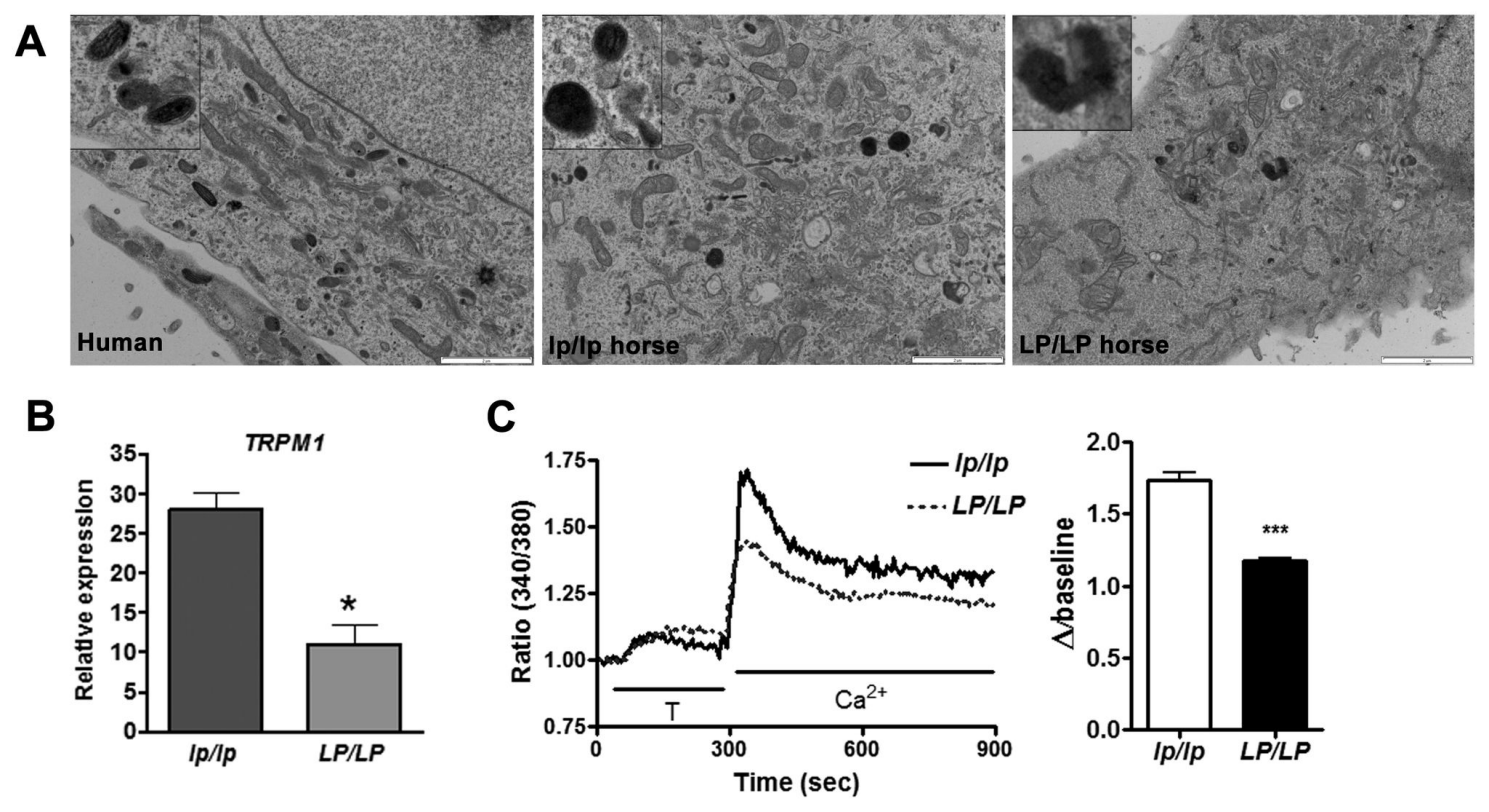
*LP/LP* and *lp/lp* equine melanocyte culture data. (A) Ultrastructure of melanocytes from human and horse epidermis at passages 3, 5, and 2 (humans, *lp/lp* and *LP/LP* respectively) were plated on glass coverslips and processed for transmission electron microscopy. Insets show melanosome morphology at higher magnification. *Scale bar: 2μm*. (B) qRT-PCR analyses of *TRPM1*, mRNA expression in *LP/LP* and wild type *lp/lp* melanocytes. *TRPM1* was differentially expressed between *LP/LP* and *lp/lp* melanocytes (*p=0.01). (C) Fluorescence ratio change in *lp/lp* and pigmented *LP/LP* melanocytes. *LP/LP* cells had significantly lower peak Ca^2+^ uptake (***p<0.001).

To begin to characterize LP melanocytes we cultured epidermal melanocytes from *lp/lp* pigmented skin and both pigmented and non-pigmented *LP/LP* skin (Figure 5). We successfully isolated and established melanocyte cultures for both the pigmented *LP/LP* and *lp/lp* skin (Figure 5). However, the non-pigmented area of the *LP/LP* horse skin did not yield any viable melanocytes that could be propagated. Interestingly, transmission electron microscopy of cultured melanocytes showed spherical melanosomes in wild type *lp/lp* melanocytes, but irregularly shaped melanosomes in *LP/LP* melanocytes (Figure 6A). *LP* genotypes of melanocytes cultures were confirmed by allele specific PCR, as described in the methods, using DNA from pelleted cultured cells. Genotypes were as expected; specifically cells isolated from the pigmented *LP/LP* sample genotyped homozygous for the LTR insertion. Differential gene expression between *LP/LP* and *lp/lp* cells, originally detected in skin samples [17], was confirmed by qRT-PCR in our cultured cells (p=0.01; Figure 6B). Furthermore, ratiometric imaging showed lower peak [Ca^2+^] uptake in *LP/LP* melanocytes compared to *lp/lp* cells, consistent with lack of TRPM1 functioning in these cells (Figure 6C).

## Discussion

**Figure 7:**
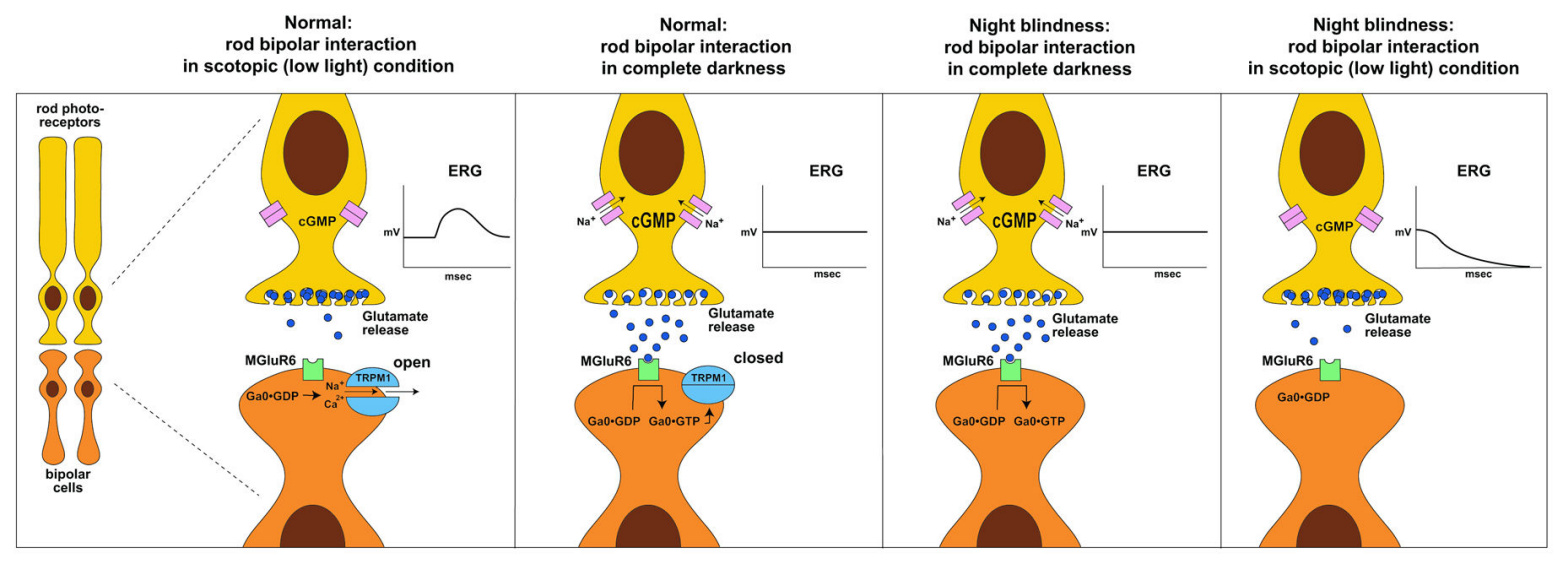
The role of TRPM1 in night vision. Night vision is controlled by ON visual pathway which comprises both retinal rod and ON-bipolar cells and involves the neurotransmitter glutamate, its receptor mGluR6, the G protein Gα_o_, TRPM1, and Ca^2+^. Photon absorption by the rod cell causes the rod cell to hyperpolarize (resulting in the “a” wave of the ERG) and decreases the concentration of glutamate being released by that cell. This leads to the opening of the calcium ion channel, TRPM1, and results in the depolarization of the ON bipolar cell (“b” wave on the ERG). In the absence of photon activation, low light conditions, the rod cell is depolarized leading to the release of glutamate and the closing of TRPM1. This results in the hyperpolarization of the ON-bipolar cell. In contrast, in *LP/LP* CSNB horses TRPM1 is unavailable to respond to changes in glutamate concentration and thus the ON-bipolar cells remain hyperpolarized in response to light. The b-wave is absent, so the ERG appears “negative” as it is only the a-wave (the rod cell response) that is detected.

CSNB in *LP* horses is explained by lack of normal TRPM1 transcription as night vision in mammals is controlled by depolarizing currents that result from the ON pathway visual system involving TRPM1 (Figure 7). Under normal dark conditions glutamate is released by the retinal rod cell which in turn binds to its G protein receptor, MGluR6, on the ON bipolar cells. The activated G protein (Gα_o_) maintains the closed state of TRPM1 cation channels and results in the hyperpolarized state of the ON-bipolar cell (denoted by a flat line ERG). Photon absorption closes cation channels that hyperpolarize the rod cell (the “a-wave” or downward deflection of the ERG) and interrupt glutamate release. Loss of the inhibitory effect of glutamate opens TRPM1 channels, resulting in the depolarization of the ON bipolar cell. The depolarization is evidenced as the “b- wave” or upward deflection on the ERG. In contrast, in *LP/LP* CSNB horses, premature poly-adenylation caused by the LTR insertion makes TRPM1 unavailable to respond to loss of glutamate release following photon absorption (Figure 7). Rod cells hyperpolarize in response to light, causing the negative a-wave, but the ON-bipolar cells cannot depolarize and no b-wave appears. The “negative ERG” is a result of the a-wave which is not eclipsed by a b-wave.

Mutations in human *nyctalopin* (*NYX*) cause X-linked forms of CSNB [36] likely by the loss of localization of TRPM1 to depolarizing cell dendrites [37,38] and thus *NYX* may explain other forms of CSNB in the horse unrelated to *LP* [39]. TRPM1 has also been localized to the synaptic ribbons of rods and melanopsin-expressing photosensitive retinal ganglion cells [40,41] suggesting yet undiscovered roles for this gene in vision. 

To date, 39 polymorphisms in *TRPM1* have been identified as causal mutations for autosomal recessive complete congenital stationary night blindness in humans [6-9,42]. While these represent a variety of mutations (missense, non-sense, frame shift, alternative splicing, and one involving a 36 kb deletion), none involve insertions or transposable elements. In humans mutations in *TRPM1* have not been associated with differences in skin pigmentation and TRPM1 knockout mice do not exhibit alterations in pigmentation [43]. Absence of expression of Gα_0_ protein and lack of coupling TRPM1 to glutamate signaling in melanocytes was proposed as a possible explanation for the lack of skin pigmentation effects in *TRPM1* mutants [44]. However, the role of this *TRPM1* structural variant in *LP* pigmentation is less clear.

TRPM1 has been shown to be involved in pigment storage and UV regulated calcium homeostasis in human melanocytes [23,24] suggesting that this gene may be involved in Ca^2+^ dependent signaling related to melanocyte proliferation and/or survival. Consistent with this, our melanocyte culture work suggests absence of melanocytes in the white patterned area. However pigmented spots in the white patterned area complicate this finding. One possibility is that the spots are caused by a somatic reversion and loss of the LTR, but this is not likely because DNA extracted from the melanocytes isolated from a pigmented spot were confirmed to have the insertion and data from RNA analysis of pigmented spots (RNA-Seq, gene expression, and 3’RACE) also show LTR driven pre-mature poly-adenylation. Thus another mechanism must explain the survival of the melanocytes resulting in the “leopard” spots of pigment. 

While the precise role that TPRM1 plays in *LP* hypopigmentation is unclear, the irregularly shaped melanosomes in *LP/LP* melanocytes (Figure 6A) suggest that pigmentation in *LP* horses could be disrupted by improper melanosome maturation. This may stimulate melanocytosis resulting in white patterned areas during development and progressive roaning during the life of the animal. Alternatively, the LTR insertion could be acting in trans, potentially producing a non-coding RNA or short peptide that affects another pigmentation gene. Interestingly, performing a bioinformatic analysis of the LTR transcript predicted regulation of retinal programmed cell death as a possible biological function for the longest ORF and RNA binding as a possible function for the second longest ORF. Since these are computational predictions, additional experimental work is needed to investigate these predicted functions and the possibility of a trans acting LTR transcript. A third hypothesis involves the miRNA encoded in intron 6 of *TRPM1*, miR-211. Recently miR-211, which is cotranscribed with TRPM1, has been shown to be expressed in normal melanocytes but reduced in melanoma [45,46]. miR211 has also been shown to regulate transforming growth factor B2R and TGF-B signaling has a role in maintenance of melanocyte stem cells [47]. Thus it is possible that premature poly-adenylation of TRPM1 in intron 1 completely abolishes miR-211 causing an increased level of TGF B signaling and resulting in either depleting the melanocyte stem cell population by induced apoptosis or inhibiting pigment production by down regulating melanogenic genes. The latter two hypotheses might help explain why CSNB is recessive while *LP* spotting is dominant. All of these hypotheses remain to be further investigated. 

We have identified a 1378 bp LTR insertion with the *TRPM1* gene that is associated with both a pigmentation phenotype (*LP*) and an eye disorder (CSNB). It is believed that LTRs may contribute to species evolution through gene regulatory changes mainly by cis-acting sequences that function as alternative promoters or novel poly-adenylation signals [29,30,48-52]. Moreover, recent retrotransposon events, involving LINE-1 elements, have been shown to disrupt normal gene function and cause disease and approximately 65 such disease causing mutations have been identified in humans alone [53]. None of those described to date involve an LTR. Thus LTR transposable element insertions creating disease phenotypes, like CSNB, are likely under negative selection. Yet, this LTR insertion was definitively detected in three ancient samples suggesting that this structural variation has been maintained in the horse population long before domestication and may have provided some selective advantage. While the exact timing of the integration of this structural variant, it’s selective advantage, and the physiological mechanism of hypopigmentation remain to be determined, we propose that this insertion disrupts normal TRPM1 transcription by premature poly-adenylation and that this abnormal transcription results in both CSNB and *LP* spotting.

## Materials and Methods

### Ethics Statement

Animals were sampled with the appropriate consent from horse owners. Those horses sacrificed were humanely euthanized following The Canadian Council on Animal Care Guidelines for Experimental Animal Use and approved by the University of Saskatchewan Animal Care Committee.

### Samples

Horses were categorized according to genotype and phenotype for *LP*, which was evaluated by coat color assessment and breeding records. Those horses utilized in the studies of CSNB were also examined by a scotopic electro-retinogram (ERG) to determine CSNB status. For the RNA sequencing experiments total RNA was isolated from three (*LP/LP*, *LP/lp*, *lp/lp*) full thickness pigmented skin biopsies from live animals and from retina (one CSNB affected *LP/LP* and one CSNB unaffected *lp/lp*). To control for changes in gene expression related to sex and pigmentation differences these samples were matched for sex (male) and base coat color (chestnut). Additional RNA samples from pigmented skin (three *LP/LP* and one *lp/lp*) and retina (two CSNB affected *LP/LP*) were utilized for 3’ RACE experiments. A total of 511 horses from seven breeds segregating for *LP* spotting and one breed not segregating for *LP* as well as 43 horses phenotyped for CSNB were utilized to genotype for the discovered insertion.

### RNA sequencing and read mapping

Total RNA was isolated from 0.5 g of skin tissue in a buffer of 4 M guanidinium isothiocyanate, 0.1 M Tris–HCl, 25 mM EDTA (pH 7.5), and 1% (v/v) 2-mercaptoethanol, followed by differential alcohol and salt precipitations as previously described [54,55]. Retinal RNA was isolated as previously described using Trizol (Invitrogen) [17]. RNA was quantified using a NanoDrop spectrophotometer (NanoDrop Technologies) and assayed for quality using a 2100 Bioanalyzer (AgilentTechnologies). Library preparation and Illumina sequencing was performed by Cornell University's Life Sciences Core Laboratory Center. RNA-Seq reads were aligned to the horse reference genome (equCab2) with Burrows-Wheeler Aligner (BWA) [56] with the default options. BAM Files generated using SAMtools [57] allowed for visualization of output alignments using the UCSC Genome Browser (http://genome-preview.ucsc.edu/cgi-bin/hgGateway?hgsid=2633482&clade=mammal&org=Horse&db=0). RNA-Seq data was submitted as studies to the Sequence Read Archive (SRA at the European Nucleotide Archive http://www.ebi.ac.uk/ena/ and can be found at http://www.ebi.ac.uk/ena/data/view/ERP001524 and http://www.ebi.ac.uk/ena/data/view/ERP001525 (Accession numbers. ERP001524 and ERP001525).

### Insertion discovery

RNA-Seq analysis identified expression of a region in intron 1 of *TRPM1* in *LP/LP* and *LP/lp* samples but not *lp/lp* skin and retina samples. This region was investigated by genomic PCR to test for structural variations by using two different sets of primers (Table S3). One primer combination (108297763F and 108299526 R) resulted in correct size product for *LP/lp* and *lp/lp* horses but only non-specific amplification in the *LP/LP* horses while amplicons involving primers spanning ECA1: 108,298,197-108,300,248 resulted in the correct size product for all genotypes, thus suggesting the presence of a structural variant not allowing for amplification from ECA1: 108, 297,763-108,298,197. Genomic sequence from the region (ECA1: 108, 297,763-108,298,197) was used for *de novo* assembly of RNA-Seq reads from the *LP/LP* CSNB retina sample using cross_match and visualized with consed (Version 20) [58]. This enabled the identification of the precise location of the insertion and identified the first 200 bp of the insertion. The full length insertion was discovered in 4 horses from 3 different breeds (2 American Miniature horses, 1 Appaloosa, and 1 Knabstrupper) by genomic PCR amplification followed by sequence walking using Sanger technology. Long range PCR using 200 ng of DNA, 2.5 pmol of each primer (108, 297,763F and108,298,197R, Table S3) and Platinum PCR SuperMix High Fidelity (Invitrogen at Life Technologies, Grand Island, NY, USA) was performed. Amplicons were gel purified using the QIAquick gel extraction kit (Qiagen Sciences, Valencia, CA, USA) and subsequently sequenced using BigDye Terminator v1.1 and ABI 310 Genetic Analyzer (Applied Biosystems, at Life Technologies, Grand Island, NY, USA) and 4 different primers (5.0 pmol) to obtain sequence information for the entire insertion (Table S3). Sequences were assembled using ContigExpress from the Vector NTI Advance 11 software package (Invitrogen at Life Technologies, Grand Island, NY, USA).

### LTR analysis

The *LP* insertion element was identified as a ERV2-1N-EC_LTR using RepeatMasker [27]. To determine the relationship of this LTR to other LTRs in the equine genome, the insertion element was used to identify all similar elements in the equine genome EquCab2.0 using LASTZ[59] based on criteria of ≥80% sequence identity and ≥90% length. An additional 202 full length LTR were identified in this fashion and their sequences, along with 50 bp of flanking sequences, were extracted from the equine genome assembly. The retrieved LTR sequences were then aligned using MUSCLE [60,61] and a phylogenetic tree computed using FastTree [62,63] with the General Time Reversible model of nucleotide substitution and Gamma20. LTR88, a mammalian LTR sequence was included to provide an outgroup for the tree. Flanking sequences were used as input for TSDscan [64] to determine if TSDs could be used to predict relative insertion age. 

### Genotyping modern horses

Whole blood or mane hair was collected by one of the authors or submitted by owners for this study. DNA from blood samples was extracted either using the Puregene whole-blood extraction kit (Qiagen Inc., Valencia, CA, USA) or Nucleon Bacc2 kit (GE Healthcare Bio-Sciences Corp., Piscataway, NJ, USA) according to the manufacturer’s protocol. Hair samples were processed using 5–7 hair bulbs according to the method described previously [65]. Standard PCR protocol using allelic specific primers allowed for genotyping of this insertion. 2.0 pmol of two forward primers 108,297,821 F (to amplify the wildtype allele 186 bp) and TRPM1 ins F” (to amplify the insert allele 225 bp) and one reverse primer (108,297,987R, Table S3) were used in the same reaction with 1 uL of DNA from hair lysis extraction or 25 ng of DNA isolated from blood, 1X PCR buffer with 2.0mM MgCl_2_, 100 µM of each dNTP, and 0.1U FastStartTaq DNA polymerase (Roche Applied Science Indianapolis, IN, USA). Association of insertion with *LP* genotype and CSNB status were analyzed by chi-square tests.

### 3’ RACE experiments

3’ RACE (Rapid Amplification of cDNA ends) ready cDNA was synthesized from 187.5 ng of total RNA (3 *LP/LP* skin and 3 *LP/LP* CSNB affected retina samples as well as 1 *lp/lp* skin and 1 *lp/lp* unaffected retina as negative controls) using 3’CDS Primer A (supplied with SMARTer^TM^ RACE cDNA amplification kit) and SMARTScribe^TM^ Reverse Transcriptase following manufactures instructions (SMARTer^TM^ RACE cDNA amplification kit Clontech Laboratories, Inc., Mountain View, CA, USA). Following cDNA synthesis 1.25 µl of diluted 3’ RACE ready cDNA was utilized as a template for 3’RACE PCR using UPM (supplied with SMARTer^TM^ RACE cDNA amplification kit) and TRPM1 lp RACE 3’ RACE F-1(Table S3). PCR was performed following manufactures instructions using the Advantage 2 PCR KIT and reducing final reaction volume to 25 µl. (Clontech Laboratories, Inc., Mountain View, CA, USA). To increase specificity, a semi-nested PCR was performed using 2.5 µl diluted PCR product (5 µl of PCR product diluted in 245 µl of Tris-EDTA buffer, pH= 8.0), UPM, TRPM1 ins TRANS R-6 (Table S3), and the same Advantage 2 PCR KIT again following manufacturer’s instructions. Products were visualized on 1% EtBr agarose gel and amplicons were gel purified using the QIAquick gel extraction kit (Qiagen Sciences, Valencia, CA, USA) and subsequently sequenced using BigDye Terminator v1.1 and ABI 310 Genetic Analyzer (Applied Biosystems, at Life Technologies, Grand Island, NY, USA). Each sample was sequenced with 4 different primers (5 pmol) to obtain full amplicon sequence information from *TRPM1* gene specific sequence (TRPM1 ins TRANS R-6) to the poly(A) tail (Table S3).

### Bioinformatics analysis of ORFs

The transcript sequence from the *LP/LP* pigmented skin sample was translated using the Sequence Manipulation Suite [66]. All identified ORFs were analyzed using BLASTP against the nr database at www.ncbi.nlm.nih.gov. These ORFs were further analyzed using the Protein Function Prediction server [33] available at http://kiharalab.org/web/pfp.php and the Extended Similarity Group server [34] available at http://kiharalab.org/web/esg.php. Only probability scores greater than or equal to 60% were retained for the predicted molecular function and biological process.

### Melanocyte Culture Experiments

#### Isolation and culture of equine melanocytes

Full thickness skin biopsies (3-6mm) were collected in DMEM medium with 2% FBS and supplemented with standard tissue culture grade antibiotic and antimycotic mixture. The tissues were sliced into small pieces and incubated in 0.25% trypsin-EDTA in 6 well plates overnight at 4°C. Next day, epidermis was separated using sterile forceps and cells were dissociated by vigorous pipetting. The tissue pieces and cells were pelleted by centrifuging at 1500 rpm for 10 minutes and re-suspended in melanocyte medium, plated in 6-well plates and cultured at 37°C with 5% CO_2_. The medium was changed 24 hours after plating and after every three days [24]. 

#### Transmission electronmicroscopy

Cells on coverslips were washed with phosphate-buffered saline, fixed with 2.5% glutaraldehyde, and processed for transmission electron microscopy. Ultrathin sections were observed and photographed using the Jeol 100 electron microscope (Tokyo, Japan) at the UW Electron Microscope Facility.

#### Quantitative RT-PCR

Total RNA was isolated using RNeasy mini kit (Qiagen, Valencia, CA). Total RNA (1–3 µg) was reverse transcribed using the SuperScript III First-Strand Synthesis system for RT-PCR (Invitrogen). Primers for *TRPM1* and *β-actin* were designed using PrimerExpress software (Applied Biosystems, Foster City, CA) and obtained from IDT Inc. (Coralville, IA). The primer sequences and the calculated amplicon length are represented in Table S4. qPCR was performed in triplicate with SYBR Green PCR core reagents (Applied Biosystems), in 20 µl of reactions containing 50-150ng cDNA and 10µM each primer. β-actin was used as the housekeeping gene control. PCR was performed using ABI 7000 and cycle parameters: denaturation at 95°C, followed by 40 cycles of 95°C for 15 s, 57°C for 15 s, and 72°C for 15 s. Relative quantification (RQ) was calculated using 2^- ΔΔC^_T_ method qRT-PCR data was analyzed according Schmittgen and Livak [67].

#### Immunofluorescence

Melanocytes were confirmed by immunostaining for the melanocyte-specific markers namely TYRP1 and MITF (Figure S4). Melanocytes grown on chamber slides (Nalge Nunc International, Naperville, IL) were fixed in 4% freshly prepared paraformaldehyde for 15 minutes and permeabilized for 5 minutes in 0.1% Triton X-100. The cells were washed, blocked for 30 minutes in 10% goat serum and incubated for 1 h with primary antibodies (anti-TYRP1 mAbTA99 at 1:1000 and anti-MITF antibody SC- 56725 at 1:100) followed by FITC-conjugated anti-mouse rabbit IgG diluted in 10% goat serum for 1 h. After washing with PBS, coverslips were mounted using a mounting medium containing anti-fade reagent (Molecular Probes, Invitrogen).

#### Calcium imaging

Melanocytes plated on glass chamber slides were washed in buffer D (153mM NaCl, 5.4mM KCl, 1.8mM CaCl_2_, 10mM N-2-hydroxy ethylpiperazine-N’-2-ethanesulfonic acid (HEPES), 25mM glucose, 0.8mM MgSO_4_, 0.9mM NaH_2_PO_4_ and 2.4mM NaHCO_3_, pH 7.4), incubated in 2 µM Fura-2 (Invitrogen) in buffer D at 37°C for 60 minutes, washed three times with buffer D without calcium and de-esterified for 30 minutes at 37°C. Cells were imaged and ratio of fluorescence emission was monitored using Nikon Ti fluorescence microscope and NIS-Elements software. In a 20x field with well separated cells (n=8-10), baseline was established in buffer D without Ca^2+^ and change in F340/380 was monitored after addition of various reagents or Ca^2+^ for a total of 15 minutes with no cell exposed to light for longer than 20 min. Results were expressed as ratio value after background fluorescence subtraction and normalization to baseline fluorescence. 

### Ancient DNA typing

DNA was extracted from eight ancient samples (Table S2) following a previously established protocol [68] in dedicated ancient DNA facilities at the Humboldt-University of Berlin, Germany and at the University of York, UK. In addition to the ancient samples, two modern samples (1 *LP*/*lp* and 1 LP/LP) were included to serve as positive controls for the enrichment strategy (Table S1). Sequencing libraries suitable for the Illumina platform were prepared following an established protocol [69] with some minor modifications: the Illumina P5 adapter sequences were modified to include a 8 bp index at the 3' end, effectively double-indexing the libraries without the need for an extra sequence read. To enrich the libraries for the DNA sequences required to detect the LTR insertion, probes were designed that overlap the boundary between TRPM1 intron 1 and the start of the insert as well as intron 1 and the end of the insert (Table S5). The target regions for capture were synthesized as biotinylated DNA oligos and pooled in equal concentrations for in-solution capture following Horn (2012) [70] in two consecutive capture rounds. After enrichment, samples were pooled in equimolar amounts and 12.5 pM of the library pool was sequenced using the MiSeq System (Illumina, San Diego, CA, USA). 100 bp paired-end sequencing was carried out; i.e. 2x101 sequencing cycles (indexing was carried out using 9 sequencing cycles) using the Illumina MiSeq Reagent Kit v2 (300 cycles). The lower concentration and poor quality of DNA in ancient samples resulted in lower sequence coverage compared to the control samples. Generated reads were cleaned from index and adapter sequences using *cutadapt* (v1.1) [71] with default parameters and different stringencies for the minimal required read length after trimming (10-30 nt). Trimmed reads were further filtered according to base quality using *fastx toolkit* (http://hannonlab.cshl.edu/fastx_toolkit/) requiring a quality score of a minimum of 30 over at least 50% of the read length. Final reads were mapped to the LTR probe sequences using Bowtie [72] to identify the *LP* insert and additionally mapped to the horse reference genome (equCab2) to confirm location in the TRPM1 intron 1. Mapped reads with a minimum length of 30 nt were required for conclusive LTR insert detection. 

## Supporting Information

Figure S1 **Investigating the expression of 3 SNPs previously identified as completely associated with *LP* and CSNB.** BAM files for each of the RNA samples utilized in this RNA-Seq experiment were aligned to the equCab2 reference genome using the UCSC Genome Browser. RNA isolated from *lp/lp* skin is denoted by 46-lp-skin, while that from LP/LP skin is denoted by 54-LP-Skin, and *LP/lp* skin is denoted by 49-LP-lp-pigmented. Retina RNA samples are denoted by 45-lp-retina (CSNB unaffected) and 54-LP retina (CSNB affected). 100 bp flanking each SNP is shown as represented by the coordinates along with any non-horse RefSeq genes. Both ECA1 g.108281765T>C (A) and ECA1 g.108288853C>T (B) are located in introns of TRPM1 as defined by human, rat and mouse RefSeq genes and this is a confirmed intron in the horse as no expression of this region was detected in either skin or retina. While ECA1g.108337089T>G (C) is also not in an exon, as no expression was detected, however no known RefSeq gene has been mapped to this region.
(TIF)

Figure S2 **RepeatMasker blast analysis summary of 1.4 kb LP/CSNB insertion.** Version 3.2.9 of RepeatMaster was utilized for this blast analysis. The LP/CSNB insertion was identified as an LTR ERV Class II.
(TIFF)

Figure S3 **The relationship of the LP/CSNB LTR insertion compared to all other equine LTRs.** The LTR in intron 1 of *TRPM1* causing both CSNB and *LP* is denoted by a red box. The inset, boxed in blue, shows a more detailed version of the branch of the tree containing the newly discovered insertion (red branch).
(TIFF)

Figure S4 **Expression of TYRP1 and MITF in melanoctyes.** Cells isolated from *lp/lp* horse cultures were compared to human cells to confirm melanocytes identity. Cells cultured on glass coverslips were fixed with paraformaldehyde and permeabilized with methanol and incubated with anti-TYRP1 and anti-MITF antibodies followed by FITC-conjugated anti-mouse IgG. Images were captured on Nikon microscope with 20x objective.
(TIF)

Table S1 **Predicted protein functions for LP insertion transcript.** Nine potential ORF of the LTR insertion transcript were investigated using the PFP and ESG protein prediction servers. GO molecular functions and biological processes with probabilities equal to or greater than 60% are reported.
(XLSX)

Table S2 ***TRPM1* insert detection in ancient DNA samples.** Genotyping was based on single molecule capture and Illumina sequencing. Data presented are MiSeq read depth after mapping with Bowtie and utilizing different filter settings for read lengths. A modern homozygote (*LP/LP*) and heterozygote (*LP/lp*) were included for validation of the data.
(XLSX)

Table S3 **Primers utilized in the discovery and the investigation of the 1.4 kb insertion causing *LP* and CSNB.** Primers were used for identification and sequencing of full length LP/CSNB insertion, genotyping for *LP* and CSNB, and for performing 3’ RACE and sequencing RACE products.
(XLSX)

Table S4 **Primers used for qRT-PCR of melanocyte cultures.**
(XLSX)

Table S5 **Probes sequences used to genotype ancient DNA for the LP/CSNB insertion.** Probes were used to enrich libraries for the target regions via hybridization capture using biotin-streptavidin binding coupled to Illumina sequencing.
(XLSX)
