# Supplementary figures and images for: Evidence for a Retroviral Insertion in TRPM1 as the Cause of Congenital Stationary Night Blindness and Leopard Complex Spotting in the Horse
Source: PLoS One. 2013 Oct 22;8(10):e78280. doi: 10.1371/journal.pone.0078280 (PMC3805535; doi:10.1371/journal.pone.0078280)

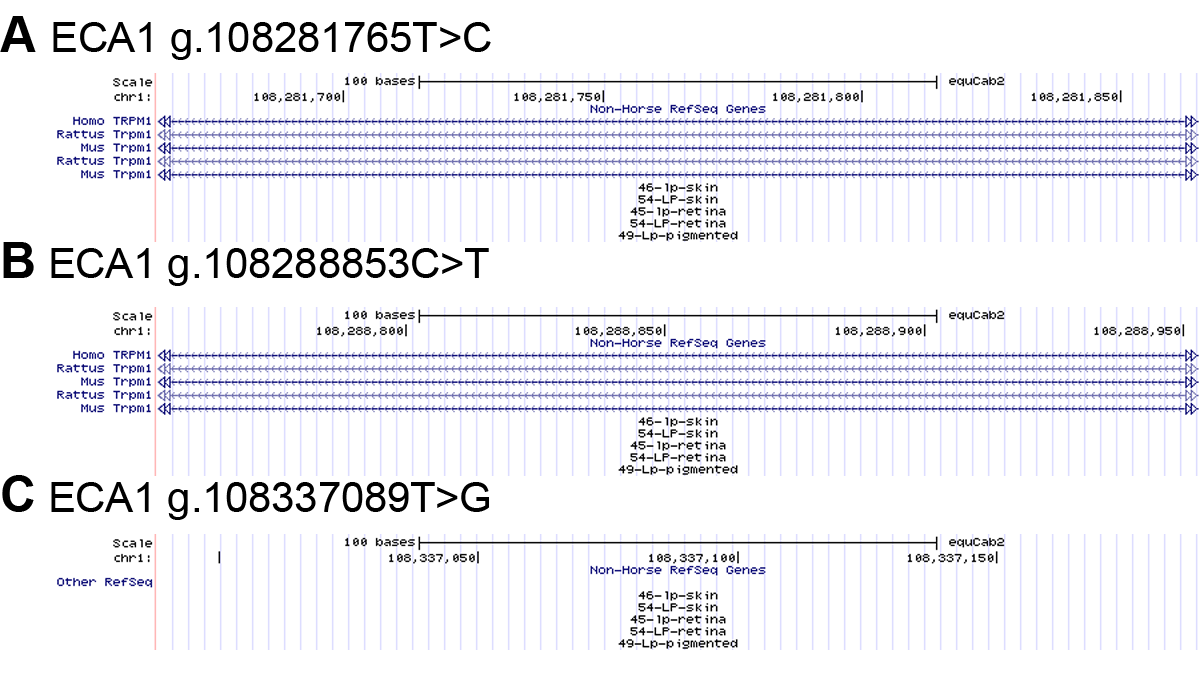

Supplement: Figure S1 — Investigating the expression of 3 SNPs previously identified as completely associated with LP and CSNB. BAM files for each of the RNA samples utilized in this RNA-Seq experiment were aligned to the equCab2 reference genome using the UCSC Genome Browser. RNA isolated from lp/lp skin is denoted by 46-lp-skin, while that from LP/LP skin is denoted by 54-LP-Skin, and LP/lp skin is denoted by 49-LP-lp-pigmented. Retina RNA samples are denoted by 45-lp-retina (CSNB unaffected) and 54-LP retina (CSNB affected). 100 bp flanking each SNP is shown as represented by the coordinates along with any non-horse RefSeq genes. Both ECA1 g.108281765T>C (A) and ECA1 g.108288853C>T (B) are located in introns of TRPM1 as defined by human, rat and mouse RefSeq genes and this is a confirmed intron in the horse as no expression of this region was detected in either skin or retina. While ECA1g.108337089T>G (C) is also not in an exon, as no expression was detected, however no known RefSeq gene has been mapped to this region. (TIF) [file pone.0078280.s001.tif]

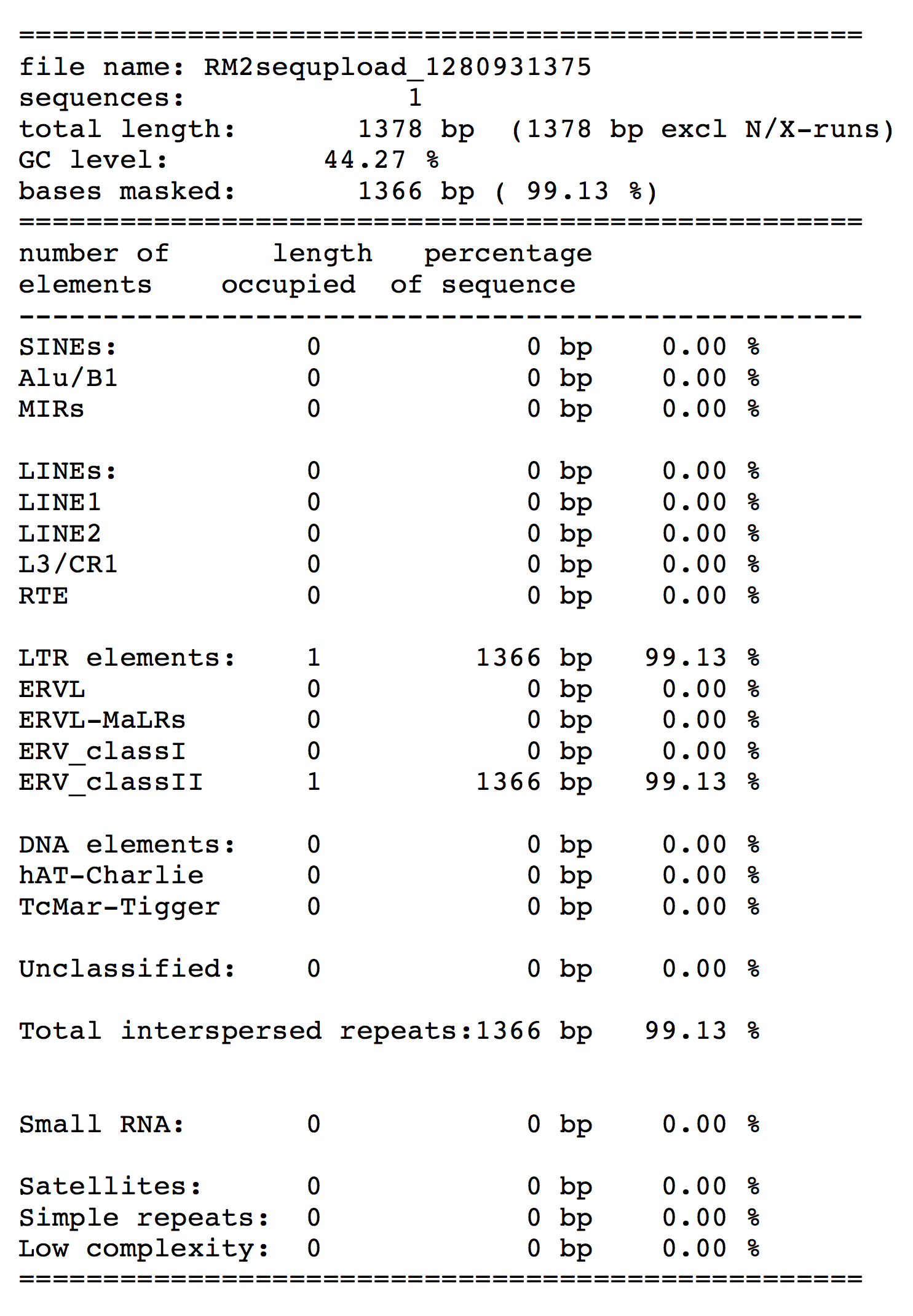

Supplement: Figure S2 — RepeatMasker blast analysis summary of 1.4 kb LP/CSNB insertion. Version 3.2.9 of RepeatMaster was utilized for this blast analysis. The LP/CSNB insertion was identified as an LTR ERV Class II. (TIFF) [file pone.0078280.s002.tiff]

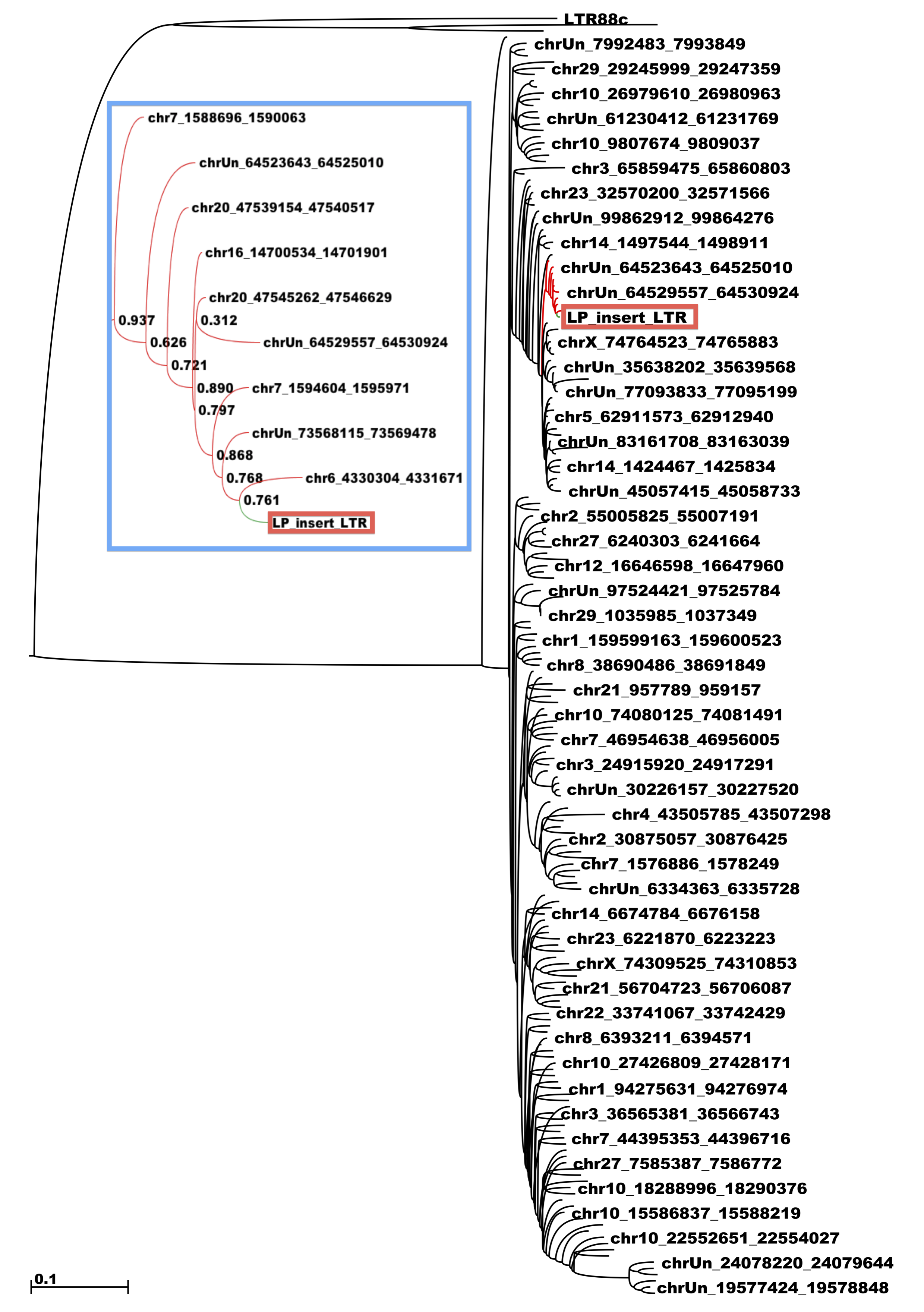

Supplement: Figure S3 — The relationship of the LP/CSNB LTR insertion compared to all other equine LTRs. The LTR in intron 1 of TRPM1 causing both CSNB and LP is denoted by a red box. The inset, boxed in blue, shows a more detailed version of the branch of the tree containing the newly discovered insertion (red branch). (TIFF) [file pone.0078280.s003.tiff]

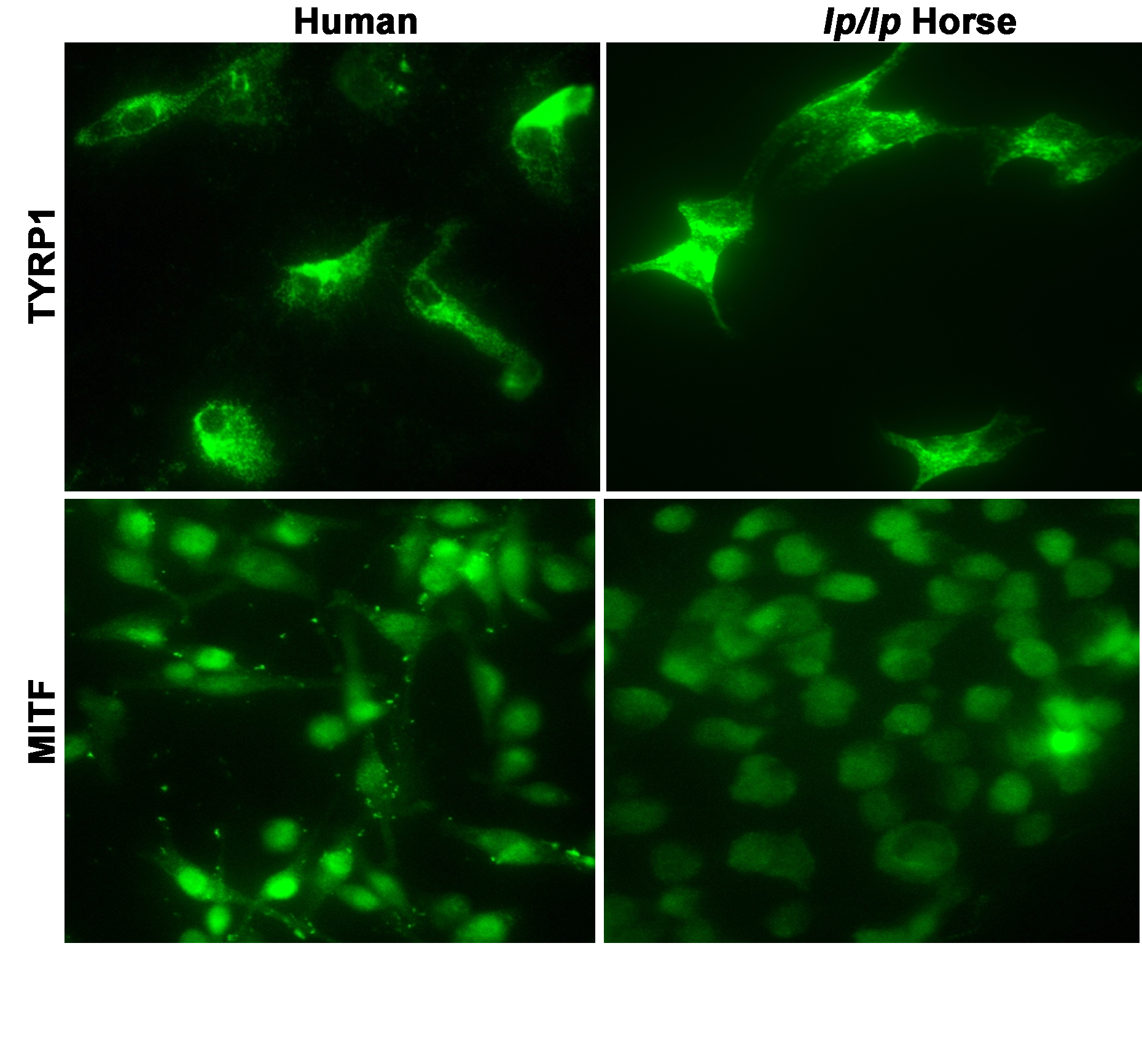

Supplement: Figure S4 — Expression of TYRP1 and MITF in melanoctyes. Cells isolated from lp/lp horse cultures were compared to human cells to confirm melanocytes identity. Cells cultured on glass coverslips were fixed with paraformaldehyde and permeabilized with methanol and incubated with anti-TYRP1 and anti-MITF antibodies followed by FITC-conjugated anti-mouse IgG. Images were captured on Nikon microscope with 20x objective. (TIF) [file pone.0078280.s004.tif]
